# Supplementary material for: Epidemiologic Factors Supporting Triage of Infected Dog Patients Admitted to a Veterinary Hospital Biological Isolation and Containment Unit
Source: Vet Sci. 2023 Mar 1;10(3):186. doi: 10.3390/vetsci10030186 (PMC10057909; doi:10.3390/vetsci10030186)
Supplement: Supplementary file 1 [file vetsci-10-00186-s001.zip › vetsci-2147670-supplementary.pdf]

Table S1: Epidemiological data from the studied population.

ID	Breed	Gender	Neuter	Age	Vaccine Status	Season	Classification	Clinical Presentation	Infectious Diagnosis	Concomitant Disorders	Hospitalization outcome	Number of Hospitalizations	Final Outcome
1	Mixed Breed	M	N	0.2	Not up to date	Warm	Suspect	Gastroenteritis		N	Discharge	1	
2	Breed	F	N	0.3	Not up to date	Warm	Suspect	Gastroenteritis		N	Discharge	1	
3	Mixed Breed	F	N	6.0		Warm	Infectious	Dermatitis	Dermatophytosis	Y	Dead	1	Dead
4	Breed	M	N	9.0	Up to date	Warm	Infectious	MDR	MDR	Y	Discharge	1	Dead
5	Breed	M	N	0.6	Not up to date	Cold	Suspect	Gastroenteritis		N	Discharge	1	
6	Mixed Breed	M	N	0.3	Not up to date	Cold	Infectious	Gastroenteritis	Parvovirosis	Y	Dead	1	Dead
7	Breed	M	N	0.3	Not up to date	Cold	Suspect	Gastroenteritis		N	Discharge	1	
8	Breed	M	N	7.0	Not up to date	Cold	Not Infectious	Leptospirosis		Y	Discharge	1	
9	Mixed Breed	M	N	0.3	Not up to date	Cold	Infectious	Gastroenteritis	Parvovirosis	N	Discharge	1	Discharge
10	Breed	M	N	0.8	Up to date	Cold	Not Infectious	Distemper		Y	Discharge	1	
11	Mixed Breed	F	N	0.6	Not up to date	Cold	Suspect	Gastroenteritis		N	Dead	1	
12	Mixed Breed	F	N	0.2	Not up to date	Cold	Infectious	Gastroenteritis	Parvovirosis	N	Discharge	1	Discharge
13	Mixed Breed	M	N	0.3	Not up to date	Cold	Infectious	Gastroenteritis	Parvovirosis	N	Discharge	1	Discharge
14	Breed	F	Y	5.0	Up to date	Cold	Infectious	Leptospirosis	Leptospirosis	N	Discharge	1	Discharge
15	Mixed Breed	M	N		Not up to date	Cold	Suspect	Gastroenteritis		N	Discharge	1	
16	Breed	F	Y	9.0	Up to date	Cold	Infectious	Dermatitis	Dermatophytosis	Y	Dead	1	Dead
17	Mixed Breed	M	N	8.0		Cold	Infectious	Dermatitis	Sarcoptic Scabies	Y	Dead	1	Dead
18	Breed	F	N	3.0	Up to date	Cold	Suspect	Distemper		Y	Dead	1	
19	Breed	F	Y	10.0	Up to date	Cold	Not Infectious	Leptospirosis		Y	Discharge	1	
20	Mixed Breed	F	N	0.7	Not up to date	Cold	Suspect	Gastroenteritis		N	Discharge	1	
21	Breed	M	N	7.0		Cold	Infectious	MDR	MDR	Y	Discharge	1	Discharge
22	Mixed Breed	F	N	1.0		Cold	Suspect	Leptospirosis		Y	Discharge	1	
23	Mixed Breed	M	N	3.0		Cold	Suspect	Leptospirosis		N	Discharge	1	
24	Breed	F	N	12.0	Up to date	Cold	Suspect	Leptospirosis		Y	Discharge	1	
25	Mixed Breed	F	N	0.6	Not up to date	Warm	Infectious	Distemper	Distemper	Y	Discharge	1	Discharge
26	Breed	M	N	9.0		Warm	Infectious	Leptospirosis	Leptospirosis	N	Dead	1	Dead
27	Mixed Breed	F	N	0.3	Not up to date	Warm	Infectious	Gastroenteritis	Parvovirosis	N	Dead	1	Dead
28	Breed	M	N	15.0	Not up to date	Warm	Not Infectious	Leptospirosis		Y	Dead	1	
29	Breed	M	N	0.2	Not up to date	Warm	Suspect	Gastroenteritis		Y	Dead	1	
30	Mixed Breed	M	N	0.2	Not up to date	Warm	Suspect	Distemper		Y	Dead	1	
31	Breed	M	N	4.0		Warm	Not Infectious	Leptospirosis		Y	Dead	1	
32	Breed	F	Y	3.0		Warm	Suspect	Gastroenteritis		N	Discharge	1	
33	Mixed Breed	F	N	12.0	Not up to date	Warm	Suspect	Leptospirosis		N	Dead	1	
34	Mixed Breed	M	N	0.5	Not up to date	Warm	Suspect	Gastroenteritis		N	Dead	1	
35	Breed	F	N	0.3		Warm	Suspect	Gastroenteritis		N	Dead	1	
36	Breed	F	N	0.3	Not up to date	Warm	Infectious	Gastroenteritis	Parvovirosis	N	Discharge	1	Discharge
37	Mixed Breed	F	N	0.6	Not up to date	Warm	Suspect	Gastroenteritis		N	Discharge	1	
38	Mixed Breed	F	N	2.0	Not up to date	Warm	Suspect	Gastroenteritis		N	Discharge	1	
39	Breed	M	N	0.8	Up to date	Warm	Suspect	Leptospirosis		N	Discharge	1	
40	Breed	F	N	0.3	Not up to date	Warm	Suspect	Gastroenteritis		N	Discharge	1	
41	Breed	M	N	0.2	Not up to date	Warm	Suspect	Gastroenteritis		N	Discharge	1	
42	Mixed Breed	F	N	4.0	Not up to date	Warm	Suspect	Leptospirosis		N	Discharge	1	
43	Mixed Breed	M	N	1.0		Warm	Suspect	Gastroenteritis		N	Discharge	1	
44	Mixed Breed	F	N	0.7	Not up to date	Warm	Infectious	Gastroenteritis	Parvovirosis	Y	Discharge	1	Discharge
45	Breed	M	N	8.0	Not up to date	Cold	Infectious	Leptospirosis	Leptospirosis	N	Dead	1	Dead
46	Mixed Breed	M	N	0.3	Not up to date	Warm	Infectious	Gastroenteritis	Parvovirosis	N	Discharge	1	Discharge
47	Mixed Breed	M	N	0.7	Not up to date	Warm	Infectious	Gastroenteritis	Parvovirosis	Y	Discharge	1	Discharge
48	Mixed Breed	F	N	0.7	Not up to date	Warm	Infectious	Gastroenteritis	Parvovirosis	N	Discharge	1	Discharge
49	Breed	M	N	0.1	Not up to date	Warm	Infectious	Gastroenteritis	Parvovirosis	Y	Dead	1	Dead
50	Mixed Breed	M	Y	0.5		Cold	Suspect	Leptospirosis		N	Dead	1	
51	Breed	F	N	0.5	Not up to date	Cold	Suspect	Gastroenteritis		N	Discharge	1	
52	Breed	F	N	0.5	Not up to date	Cold	Suspect	Gastroenteritis		N	Discharge	1	
53	Breed	M	N	3.0	Up to date	Cold	Infectious	Leptospirosis	Leptospirosis	Y	Discharge	1	Discharge
54	Breed	M	N	0.3	Not up to date	Cold	Infectious	Distemper	Distemper	N	Discharge	3	Discharge
55	Breed	M	N	8.0	Up to date	Cold	Suspect	Leptospirosis		Y	Dead	1	
56	Mixed Breed	F	Y	10.0	Up to date	Cold	Not Infectious	Leptospirosis		Y	Discharge	1	
57	Breed	M	N	6.0	Up to date	Cold	Infectious	MDR	MDR	Y	Dead	1	Dead
58	Mixed Breed	F	N	8.0	Up to date	Cold	Infectious	Leptospirosis	Leptospirosis	Y	Discharge	1	Discharge
59	Mixed Breed	M	N	0.3	Not up to date	Cold	Suspect	Gastroenteritis		N	Dead	1	
60	Mixed Breed	F	N	8.0	Not up to date	Cold	Infectious	Leptospirosis	Leptospirosis	N	Discharge	1	Dead
61	Mixed Breed	F	N	3.0	Not up to date	Cold	Infectious	Leptospirosis	Leptospirosis	N	Discharge	1	Discharge
62	Breed	M	N	12.0	Up to date	Cold	Suspect	Leptospirosis		N	Dead	1	
63	Breed	M	N	6.0	Up to date	Cold	Infectious	Leptospirosis	Leptospirosis	Y	Dead	1	Dead
64	Breed	F	N	11.0		Cold	Not Infectious	Leptospirosis		Y	Dead	1	
65	Mixed Breed	M	N	0.5	Not up to date	Cold	Infectious	Gastroenteritis	Parvovirosis	N	Discharge	1	Discharge
66	Mixed Breed	M	N	0.3		Cold	Suspect	Gastroenteritis		N	Dead	1	
67	Breed	M	N	9.0	Up to date	Cold	Infectious	MDR	MDR	Y	Discharge	1	Discharge
68	Mixed Breed	F	N	1.0	Not up to date	Cold	Suspect	Gastroenteritis		N	Discharge	1	
69	Breed	F	N	1.0	Not up to date	Warm	Suspect	Dermatitis		N	Discharge	1	Discharge
70	Breed	F	N	0.4	Up to date	Warm	Infectious	MDR	MDR	N	Discharge	1	Discharge
71	Breed	M	N	1.0	Not up to date	Warm	Infectious	Gastroenteritis	Parvovirosis	N	Discharge	1	Discharge
72	Mixed Breed	F	N	0.3	Not up to date	Warm	Infectious	Gastroenteritis	Parvovirosis	N	Discharge	1	Discharge
73	Mixed Breed	M	N	0.3	Not up to date	Warm	Infectious	Gastroenteritis	Parvovirosis	Y	Discharge	1	Discharge
74	Mixed Breed	M	N	5.0		Warm	Infectious	Distemper	Distemper	Y	Dead	1	Dead
75	Mixed Breed	M	N	0.2	Not up to date	Warm	Infectious	Gastroenteritis	Parvovirosis	N	Discharge	1	Discharge
76	Mixed Breed	F	N	0.2	Not up to date	Warm	Infectious	Gastroenteritis	Parvovirosis	N	Discharge	1	Discharge
77	Mixed Breed	M	N	0.2	Not up to date	Warm	Infectious	Gastroenteritis	Parvovirosis	N	Discharge	1	Discharge
78	Mixed Breed	M	N	0.4	Not up to date	Warm	Infectious	Gastroenteritis	Parvovirosis	Y	Discharge	1	Discharge
79	Breed	F	N	1.0	Not up to date	Warm	Infectious	Gastroenteritis	Parvovirosis	N	Discharge	1	Discharge
80	Breed	M	N	1.0	Not up to date	Warm	Infectious	Gastroenteritis	Parvovirosis	Y	Discharge	1	Discharge
81	Mixed Breed	F	N	0.3	Not up to date	Warm	Suspect	Gastroenteritis		N	Discharge	1	
82	Mixed Breed	M	N	0.3		Warm	Suspect	Gastroenteritis		N	Discharge	1	
83	Mixed Breed	F	N	2.0		Warm	Infectious	Gastroenteritis	Parvovirosis	N	Discharge	1	Discharge
84	Mixed Breed	F	N	0.3		Warm	Infectious	Gastroenteritis	Parvovirosis	N	Discharge	1	Discharge
85	Mixed Breed	M	N	1.0		Warm	Infectious	Gastroenteritis	Parvovirosis	N	Discharge	1	Discharge
86	Mixed Breed	F	N	1.0		Warm	Infectious	Gastroenteritis	Parvovirosis	N	Discharge	1	Discharge
87	Mixed Breed	M	N	0.5	Not up to date	Warm	Infectious	Gastroenteritis	Parvovirosis	Y	Discharge	1	Discharge
88	Mixed Breed	M	N	6.0	Not up to date	Warm	Suspect	Leptospirosis		N	Dead	1	
89	Mixed Breed	F	Y	0.5		Warm	Infectious	Leptospirosis	Leptospirosis	N	Discharge	1	Discharge
90	Mixed Breed	M	N	5.0	Not up to date	Warm	Infectious	Gastroenteritis	Parvovirosis	N	Discharge	1	Discharge
91	Breed	M	N	0.8	Not up to date	Warm	Infectious	Gastroenteritis	Parvovirosis	Y	Discharge	1	Discharge
92	Mixed Breed	F	N	0.4		Warm	Infectious	Gastroenteritis	Parvovirosis	N	Discharge	1	Discharge
93	Breed	F	N	6.0	Not up to date	Warm	Infectious	Distemper	Distemper	N	Discharge	1	Discharge
94	Breed	M	N	9.0		Warm	Infectious	Distemper	Distemper	N	Dead	1	Dead
95	Mixed Breed	M	N	1.0	Not up to date	Cold	Infectious	Gastroenteritis	Parvovirosis	N	Dead	1	Dead
96	Breed	F	N	0.8	Up to date	Cold	Infectious	Dermatitis	Dermatophytosis	Y	Discharge	1	Discharge
97	Mixed Breed	M	N	9.0	Not up to date	Cold	Infectious	Leptospirosis	Leptospirosis	Y	Dead	1	Dead
98	Mixed Breed	M	N	0.2	Not up to date	Cold	Suspect	Gastroenteritis		N	Discharge	1	
99	Breed	F	N	8.0	Not up to date	Cold	Not Infectious	Leptospirosis		Y	Discharge	1	
100	Mixed Breed	M	N	0.3	Not up to date	Cold	Suspect	Gastroenteritis		Y	Discharge	1	
101	Mixed Breed	F	N	0.6		Cold	Suspect	Gastroenteritis		N	Discharge	1	
102	Breed	F	N	3.0	Not up to date	Cold	Suspect	Leptospirosis		N	Dead	1	
103	Mixed Breed	M	N	14.0	Not up to date	Cold	Suspect	Others		Y	Discharge	1	Discharge
104	Breed	M	N	0.3	Up to date	Cold	Suspect	Distemper		N	Discharge	1	
105	Breed	F	N	3.0	Not up to date	Cold	Infectious	Leptospirosis	Leptospirosis	Y	Dead	1	Dead
106	Mixed Breed	F	N	0.3		Cold	Infectious	Gastroenteritis	Parvovirosis	N	Dead	1	Dead
107	Breed	F	N	0.4	Not up to date	Cold	Infectious	Distemper	Distemper	Y	Discharge	3	Dead
108	Breed	M	Y	2.0	Not up to date	Cold	Suspect	Gastroenteritis		N	Discharge	1	
109	Mixed Breed	F	N	2.0		Cold	Suspect	Gastroenteritis		N	Discharge	1	
110	Mixed Breed	M	N	2.0		Cold	Infectious	Distemper	Distemper	N	Discharge	2	Dead

111	Breed	M	N	1.0	Up to date	Cold	Not Infectious	Leptospirosis		N	Discharge	1	
112	Mixed Breed	M	N	5.0		Cold	Infectious	Respiratory	Aspergillus rhinitis	N	Discharge	1	Discharge
113	Breed	M	N	8.0		Cold	Infectious	Respiratory	Infectious Tracheobronchitis	Y	Dead	1	Dead
114	Mixed Breed	M	N	1.0		Cold	Infectious	Distemper	Distemper	N	Dead	1	Dead
115	Breed	M	Y	0.9	Up to date	Cold	Suspect	Gastroenteritis		N	Discharge	1	
116	Breed	M	Y	8.0	Not up to date	Cold	Infectious	MDR	MDR	Y	Discharge	2	Dead
117	Breed	F	N	7.0	Up to date	Cold	Infectious	MDR	MDR	Y	Discharge	1	Discharge
118	Mixed Breed	F	N	0.2	Not up to date	Cold	Infectious	Distemper	Distemper	N	Dead	1	Dead
119	Breed	F	N	9.0	Up to date	Cold	Infectious	Leptospirosis	Leptospirosis	N	Discharge	1	Discharge
120	Mixed Breed	M	N	2.0		Cold	Infectious	Distemper	Distemper	Y	Dead	1	Dead
121	Breed	F	Y	8.0	Not up to date	Cold	Infectious	Dermatitis	Dermatophytosis	Y	Discharge	1	Discharge
122	Breed	M	N	2.0	Not up to date	Cold	Suspect	Distemper		N	Discharge	1	Discharge
123	Mixed Breed	M	N	0.2	Not up to date	Cold	Suspect	Gastroenteritis		N	Discharge	1	
124	Mixed Breed	M	N	1.0	Not up to date	Cold	Infectious	Leptospirosis	Leptospirosis	Y	Discharge	1	Discharge
125	Mixed Breed	F	Y	5.0	Up to date	Cold	Infectious	Leptospirosis	Leptospirosis	N	Discharge	1	Discharge
126	Breed	M	N	3.0	Not up to date	Cold	Not Infectious	Distemper		Y	Discharge	1	
127	Mixed Breed	F	Y	0.8	Up to date	Cold	Not Infectious	Distemper		Y	Discharge	1	
128	Breed	M	N	9.0	Not up to date	Cold	Suspect	Gastroenteritis		Y	Discharge	1	
129	Mixed Breed	M	N	5.0	Not up to date	Cold	Infectious	Distemper	Distemper	N	Dead	1	Dead
130	Mixed Breed	M	Y	2.0		Cold	Suspect	Distemper		N	Discharge	1	
131	Mixed Breed	F	N	1.0	Not up to date	Cold	Infectious	Distemper	Distemper	N	Discharge	2	Discharge
132	Breed	M	N	3.0	Up to date	Cold	Not Infectious	Leptospirosis		Y	Discharge	1	
133	Mixed Breed	F	N	5.0	Not up to date	Warm	Not Infectious	Leptospirosis		Y	Dead	1	
134	Breed	M	N	0.8	Up to date	Warm	Infectious	Distemper	Distemper	N	Discharge	1	Discharge
135	Mixed Breed	M	N	7.0	Not up to date	Warm	Infectious	Leptospirosis	Leptospirosis	Y	Dead	1	Dead
136	Mixed Breed	F	N	0.3	Not up to date	Warm	Infectious	Others	Adenovirus	N	Discharge	1	Discharge
137	Breed	M	N	0.3	Not up to date	Warm	Infectious	Gastroenteritis	Parvovirosis	N	Discharge	1	Discharge
138	Breed	M	N	0.2	Not up to date	Warm	Infectious	Gastroenteritis	Parvovirosis	N	Discharge	1	Discharge
139	Mixed Breed	M	N	0.3	Not up to date	Warm	Infectious	Distemper	Distemper	N	Discharge	1	Discharge
140	Breed	M	N	2.0	Up to date	Warm	Suspect	Dermatitis		N	Discharge	1	
141	Mixed Breed	M	N	13.0		Warm	Not Infectious	Leptospirosis		N	Dead	1	
142	Mixed Breed	M	N	11.0	Up to date	Warm	Suspect	Leptospirosis		N	Dead	1	
143	Mixed Breed	M	N	3.0	Not up to date	Warm	Infectious	Distemper	Distemper	N	Dead	1	Dead
144	Breed	M	N	11.0	Not up to date	Warm	Infectious	MDR	MDR	Y	Dead	1	Dead
145	Breed	M	N	8.0	Not up to date	Warm	Not Infectious	Leptospirosis		Y	Discharge	1	
146	Breed	M	N	0.3	Not up to date	Warm	Infectious	Others	Adenovirus	Y	Discharge	1	Discharge
147	Breed	M	N	3.0	Not up to date	Warm	Suspect	Gastroenteritis		N	Discharge	1	
148	Mixed Breed	M	N	12.0		Warm	Suspect	MDR		Y	Discharge	1	Discharge
149	Breed	F	N	0.2	Not up to date	Warm	Infectious	Gastroenteritis	Parvovirosis	N	Discharge	1	Discharge
150	Mixed Breed	M	N	2.0		Warm	Infectious	Respiratory	Streptococcus Rhinitis	Y	Discharge	1	Discharge
151	Mixed Breed	M	N	0.9		Warm	Suspect	Distemper		N	Discharge	1	
152	Mixed Breed	F	N	13.0	Not up to date	Warm	Infectious	Dermatitis	Dermatophytosis	Y	Dead	1	Dead
153	Mixed Breed	F	N	0.2	Not up to date	Warm	Suspect	Gastroenteritis		N	Discharge	1	
154	Breed	M	N	1.0	Up to date	Warm	Not Infectious	Leptospirosis		Y	Discharge	1	
155	Breed	F	N	7.0	Not up to date	Warm	Infectious	Leptospirosis	Leptospirosis	N	Discharge	1	Dead
156	Mixed Breed	M	N	1.0	Not up to date	Warm	Suspect	Gastroenteritis		N	Discharge	1	
157	Mixed Breed	M	N	0.6	Not up to date	Warm	Suspect	Gastroenteritis		N	Discharge	1	
158	Breed	M	N	0.2	Not up to date	Warm	Infectious	Gastroenteritis	Parvovirosis	N	Discharge	1	Discharge
159	Breed	M	N	0.8	Not up to date	Warm	Infectious	Gastroenteritis	Parvovirosis	N	Discharge	1	Discharge
160	Breed	F	N	0.2	Not up to date	Warm	Suspect	Gastroenteritis		N	Discharge	1	
161	Mixed Breed	F	N	15.0	Not up to date	Warm	Not Infectious	Distemper		Y	Discharge	1	
162	Mixed Breed	M	N	0.6	Not up to date	Warm	Infectious	MDR	MDR	N	Discharge	1	Discharge
163	Breed	M	N	12.0	Up to date	Warm	Suspect	Leptospirosis		Y	Discharge	1	
164	Mixed Breed	F	N	4.0	Not up to date	Warm	Infectious	Leptospirosis	Leptospirosis	N	Dead	1	Dead
165	Breed	M	N	0.8	Not up to date	Warm	Infectious	Gastroenteritis	Parvovirosis	N	Discharge	1	Discharge
166	Breed	F	Y	12.0		Warm	Suspect	Leptospirosis		Y	Discharge	1	
167	Mixed Breed	F	Y	4.0	Not up to date	Warm	Suspect	Leptospirosis		Y	Discharge	1	
168	Mixed Breed	F	N	8.0	Not up to date	Warm	Infectious	Leptospirosis	Leptospirosis	Y	Dead	1	Dead
169	Breed	F	N	0.4	Up to date	Warm	Infectious	Others	Adenovirus	N	Discharge	1	Discharge
170	Breed	F	N	0.3	Not up to date	Warm	Infectious	Others	Adenovirus	N	Discharge	1	Discharge
171	Mixed Breed	M	N	0.2	Not up to date	Warm	Infectious	Gastroenteritis	Parvovirosis	N	Discharge	1	Discharge
172	Breed	M	N	3.0	Up to date	Warm	Infectious	MDR	MDR	Y	Discharge	1	Discharge
173	Breed	F	N	3.0	Not up to date	Warm	Infectious	Leptospirosis	Leptospirosis	N	Discharge	1	Discharge
174	Mixed Breed	F	N	0.2	Not up to date	Warm	Infectious	Gastroenteritis	Parvovirosis	Y	Discharge	1	Discharge
175	Mixed Breed	M	N	12.0	Not up to date	Warm	Not Infectious	Leptospirosis		Y	Discharge	1	
176	Breed	M	N	0.2	Not up to date	Warm	Infectious	Gastroenteritis	Parvovirosis	N	Discharge	1	Discharge
177	Mixed Breed	M	N	9.0	Not up to date	Cold	Suspect	Distemper		N	Discharge	1	
178	Breed	F	Y	8.0	Not up to date	Cold	Infectious	Leptospirosis	Leptospirosis	N	Dead	1	Dead
179	Breed	M	N	0.2	Not up to date	Cold	Infectious	Gastroenteritis	Parvovirosis	Y	Discharge	1	Discharge
180	Breed	M	N	0.2	Not up to date	Cold	Infectious	Gastroenteritis	Parvovirosis	N	Dead	1	Dead
181	Breed	M	N	5.0	Up to date	Cold	Not Infectious	Leptospirosis		Y	Dead	1	
182	Mixed Breed	M	N			Cold	Suspect	Others		Y	Discharge	1	Discharge
183	Breed	M	N	4.0	Up to date	Cold	Infectious	Respiratory	Infectious Tracheobronchitis	Y	Discharge	1	Discharge
184	Breed	F	N	0.3	Not up to date	Cold	Infectious	Gastroenteritis	Parvovirosis	N	Discharge	1	Discharge
185	Breed	M	N	0.3	Not up to date	Cold	Infectious	Gastroenteritis	Parvovirosis	N	Discharge	1	Discharge
186	Mixed Breed	F	N	4.0	Not up to date	Cold	Infectious	Leptospirosis	Leptospirosis	N	Dead	1	Dead
187	Breed	F	N	6.0	Not up to date	Cold	Infectious	Leptospirosis	Leptospirosis	Y	Discharge	1	Discharge
188	Breed	M	N	0.3	Not up to date	Cold	Infectious	Distemper	Distemper	N	Discharge	1	Dead
189	Breed	F	N	4.0	Up to date	Cold	Infectious	Distemper	Distemper	Y	Dead	1	Dead
190	Breed	F	N	1.0	Not up to date	Cold	Infectious	Leptospirosis	Leptospirosis	N	Discharge	1	Discharge
191	Mixed Breed	M	N	0.9	Not up to date	Cold	Infectious	Distemper	Distemper	Y	Discharge	2	Dead
192	Breed	M	N	0.3	Not up to date	Cold	Suspect	Distemper		N	Discharge	1	
193	Breed	M	N	0.3	Not up to date	Cold	Infectious	Distemper	Distemper	N	Dead	1	Dead
194	Breed	M	N	1.0	Not up to date	Cold	Infectious	Leptospirosis	Leptospirosis	N	Discharge	1	Discharge
195	Breed	F	Y	12.0	Not up to date	Cold	Not Infectious	Leptospirosis		Y	Discharge	1	
196	Breed	F	N	5.0	Up to date	Cold	Not Infectious	Leptospirosis		Y	Discharge	1	
197	Breed	M	N	0.7		Cold	Infectious	Respiratory	Infectious Tracheobronchitis	N	Discharge	1	Discharge
198	Breed	F	N	3.0	Up to date	Cold	Infectious	Leptospirosis	Leptospirosis	N	Discharge	1	Discharge
199	Mixed Breed	F	N			Cold	Suspect	Distemper		N	Discharge	1	
200	Breed	F	N	0.3		Cold	Infectious	Gastroenteritis	Parvovirosis	N	Discharge	1	Discharge
201	Mixed Breed	F	Y	9.0	Not up to date	Cold	Not Infectious	Leptospirosis		Y	Discharge	1	
202	Breed	M	N	0.2	Not up to date	Cold	Infectious	Gastroenteritis	Parvovirosis	N	Discharge	1	Discharge
203	Breed	M	N	8.0	Up to date	Cold	Not Infectious	Leptospirosis		N	Discharge	1	
204	Mixed Breed	M	N	10.0	Not up to date	Cold	Not Infectious	Distemper		Y	Discharge	1	
205	Mixed Breed	F	Y	8.0	Not up to date	Cold	Not Infectious	Leptospirosis		Y	Discharge	1	
206	Breed	F	N	0.8	Up to date	Cold	Suspect	Leptospirosis		N	Discharge	1	
207	Breed	M	N	15.0		Cold	Infectious	Distemper	Distemper	Y	Dead	1	Dead
208	Breed	F	N	0.3	Not up to date	Cold	Infectious	Gastroenteritis	Parvovirosis	Y	Discharge	1	Discharge
209	Breed	M	Y	12.0	Up to date	Cold	Infectious	MDR	MDR	Y	Discharge	2	Discharge
210	Mixed Breed	M	N	0.8	Up to date	Cold	Not Infectious	Distemper		N	Discharge	1	
211	Mixed Breed	F	N	2.0	Not up to date	Cold	Suspect	Others		Y	Discharge	1	Discharge
212	Mixed Breed	F	N	14.0	Not up to date	Cold	Infectious	MDR	MDR	Y	Dead	1	Dead
213	Breed	M	N	0.3	Not up to date	Cold	Infectious	Leptospirosis	Leptospirosis	N	Discharge	1	Discharge
214	Breed	M	N	0.7	Up to date	Cold	Infectious	Leptospirosis	Leptospirosis	N	Discharge	1	Discharge
215	Breed	M	N	8.0	Not up to date	Cold	Infectious	Leptospirosis	Leptospirosis	N	Dead	1	Dead

216	Breed	M	N	12.0	Not up to date	Cold	Not Infectious	Leptospirosis		Y	Discharge	1	
217	Mixed Breed	M	N	9.0	Not up to date	Cold	Infectious	Distemper	Distemper	N	Dead	1	Dead
218	Mixed Breed	M	N	4.0	Not up to date	Cold	Infectious	Gastroenteritis	Parvovirosis	N	Discharge	1	Discharge
219	Mixed Breed	M	N	14.0	Not up to date	Cold	Suspect	Leptospirosis		Y	Dead	1	
220	Mixed Breed	F	N	5.0	Up to date	Cold	Not Infectious	Leptospirosis		Y	Discharge	1	
221	Mixed Breed	M	N	0.1	Not up to date	Cold	Infectious	Others	Herpesvirus	Y	Discharge	1	Discharge
222	Breed	M	N	0.5	Not up to date	Cold	Infectious	Gastroenteritis	Parvovirosis	N	Discharge	1	Discharge
223	Mixed Breed	M	N	0.1	Not up to date	Cold	Suspect	Gastroenteritis		N	Discharge	1	
224	Mixed Breed	F	N	0.3	Not up to date	Cold	Suspect	Distemper		Y	Discharge	1	
225	Breed	M	N	5.0	Not up to date	Cold	Infectious	Leptospirosis	Leptospirosis	N	Discharge	1	Discharge
226	Breed	M	N	2.0	Not up to date	Cold	Not Infectious	Distemper		N	Discharge	1	
227	Mixed Breed	M	N	13.0	Not up to date	Warm	Not Infectious	Leptospirosis		Y	Discharge	1	
228	Breed	M	N	8.0	Not up to date	Warm	Not Infectious	Leptospirosis		Y	Discharge	1	
229	Mixed Breed	M	N	0.9	Not up to date	Warm	Infectious	Leptospirosis	Leptospirosis	N	Discharge	2	Discharge
230	Mixed Breed	F	Y	12.0	Up to date	Warm	Infectious	Dermatitis	Dermatophytosis	Y	Discharge	1	Discharge
231	Breed	M	N	14.0	Not up to date	Warm	Not Infectious	Leptospirosis		Y	Discharge	1	
232	Mixed Breed	F	N	9.0	Not up to date	Warm	Infectious	Leptospirosis	Leptospirosis	N	Dead	1	Dead
233	Mixed Breed	M	Y	11.0	Up to date	Warm	Not Infectious	Leptospirosis		Y	Dead	1	
234	Mixed Breed	M	N	4.0	Up to date	Warm	Infectious	Leptospirosis	Leptospirosis	N	Discharge	1	Discharge
235	Breed	F	Y	5.0	Not up to date	Warm	Suspect	Distemper		N	Dead	1	Dead
236	Breed	F	N	0.2	Not up to date	Warm	Infectious	Gastroenteritis	Parvovirosis	N	Dead	1	Dead
237	Breed	F	N	6.0	Not up to date	Warm	Not Infectious	Leptospirosis		Y	Discharge	1	
238	Mixed Breed	M	N	0.2	Not up to date	Warm	Infectious	Gastroenteritis	Parvovirosis	N	Discharge	1	Discharge
239	Breed	M	N	7.0	Up to date	Warm	Infectious	Leptospirosis	Leptospirosis	Y	Discharge	1	Discharge
240	Mixed Breed	M	N	0.2	Not up to date	Warm	Infectious	Gastroenteritis	Parvovirosis	Y	Discharge	1	Discharge
241	Breed	M	N	9.0	Not up to date	Warm	Infectious	Leptospirosis	Leptospirosis	Y	Dead	1	Dead
242	Breed	M	N	10.0	Not up to date	Warm	Not Infectious	Leptospirosis		Y	Discharge	1	
243	Breed	M	N	0.4	Not up to date	Warm	Infectious	Distemper	Distemper	N	Dead	1	Dead
244	Mixed Breed	F	Y	4.0	Not up to date	Warm	Not Infectious	Leptospirosis		Y	Discharge	1	
245	Mixed Breed	M	N	0.5	Not up to date	Warm	Suspect	Leptospirosis		N	Discharge	1	
246	Mixed Breed	M	N	0.2	Not up to date	Warm	Infectious	Gastroenteritis	Parvovirosis	N	Discharge	1	Discharge
247	Breed	F	N	13.0	Not up to date	Warm	Not Infectious	Leptospirosis		Y	Discharge	1	
248	Mixed Breed	M	Y	8.0	Not up to date	Warm	Not Infectious	Leptospirosis		Y	Dead	1	
249	Mixed Breed	M	N	10.0	Not up to date	Warm	Suspect	Gastroenteritis		N	Discharge	1	
250	Mixed Breed	M	N	10.0		Warm	Suspect	Distemper		Y	Discharge	1	
251	Breed	M	N	6.0	Not up to date	Warm	Suspect	Leptospirosis		N	Dead	1	Dead
252	Mixed Breed	M	Y	6.0	Not up to date	Warm	Not Infectious	Distemper		Y	Discharge	1	
253	Mixed Breed	F	N	2.0	Not up to date	Warm	Suspect	Gastroenteritis		N	Discharge	1	
254	Mixed Breed	M	N	0.2	Not up to date	Warm	Suspect	Gastroenteritis		N	Discharge	1	
255	Mixed Breed	M	N	9.0	Not up to date	Warm	Suspect	Leptospirosis		N	Dead	1	
256	Mixed Breed	M	N	0.3	Not up to date	Warm	Infectious	Gastroenteritis	Parvovirosis	Y	Discharge	2	Dead
257	Breed	F	N	0.2	Not up to date	Warm	Infectious	Gastroenteritis	Parvovirosis	N	Discharge	1	Discharge
258	Breed	M	N	3.0	Not up to date	Warm	Infectious	Leptospirosis	Leptospirosis	N	Dead	1	Dead
259	Mixed Breed	M	N	14.0	Not up to date	Warm	Suspect	Distemper		N	Dead	1	
260	Mixed Breed	M	N	10.0	Not up to date	Warm	Not Infectious	Leptospirosis		N	Discharge	1	
261	Breed	F	Y	6.0	Not up to date	Warm	Suspect	Leptospirosis		Y	Dead	1	
262	Breed	M	N	0.8	Not up to date	Warm	Infectious	Gastroenteritis	Parvovirosis	N	Dead	1	Dead
263	Mixed Breed	M	N	0.3	Not up to date	Warm	Infectious	Gastroenteritis	Parvovirosis	Y	Discharge	1	Discharge
264	Breed	M	N	10.0	Not up to date	Warm	Infectious	MDR	MDR	Y	Discharge	1	Discharge
265	Mixed Breed	M	N	0.3	Not up to date	Warm	Infectious	Gastroenteritis	Parvovirosis	N	Discharge	1	Discharge
266	Breed	F	N	0.2	Not up to date	Warm	Infectious	Gastroenteritis	Parvovirosis	N	Discharge	1	Discharge
267	Mixed Breed	F	N	0.5	Not up to date	Warm	Infectious	Gastroenteritis	Parvovirosis	N	Dead	1	Dead
268	Mixed Breed	F	Y	9.0	Up to date	Warm	Suspect	Leptospirosis		Y	Discharge	1	Discharge
269	Mixed Breed	M	N	8.0	Up to date	Warm	Infectious	Leptospirosis	Leptospirosis	Y	Discharge	1	Discharge
270	Breed	M	N	7.0		Warm	Infectious	MDR	MDR	N	Discharge	2	Discharge
271	Mixed Breed	F	N	11.0	Not up to date	Warm	Not Infectious	Leptospirosis		Y	Discharge	1	
272	Breed	F	N	0.3	Not up to date	Warm	Infectious	Gastroenteritis	Parvovirosis	N	Discharge	1	Discharge
273	Breed	F	Y	4.0	Not up to date	Warm	Suspect	Leptospirosis		N	Discharge	1	Discharge
274	Breed	M	N	0.4	Not up to date	Warm	Suspect	Distemper		N	Discharge	1	
275	Mixed Breed	F	Y	9.0	Not up to date	Warm	Not Infectious	Leptospirosis		Y	Discharge	1	
276	Breed	F	N	0.3	Not up to date	Cold	Infectious	Gastroenteritis	Parvovirosis	N	Discharge	1	Discharge
277	Breed	F	N	0.6	Up to date	Warm	Not Infectious	Distemper		N	Discharge	1	
278	Breed	M	N	5.0	Up to date	Warm	Suspect	Gastroenteritis		Y	Discharge	1	
279	Mixed Breed	F	Y	13.0	Up to date	Warm	Suspect	Others		N	Discharge	1	Discharge
280	Breed	F	Y	11.0	Not up to date	Warm	Infectious	MDR	MDR	Y	Dead	1	Dead
281	Breed	F	N	0.2	Not up to date	Warm	Suspect	Gastroenteritis		N	Discharge	1	
282	Breed	M	N	3.0	Not up to date	Warm	Not Infectious	Leptospirosis		Y	Dead	1	
283	Breed	M	N	9.0	Not up to date	Cold	Not Infectious	Leptospirosis		N	Discharge	1	
284	Breed	M	N	2.0	Up to date	Cold	Suspect	Others		Y	Discharge	1	Discharge
285	Breed	F	N	0.4		Cold	Infectious	Gastroenteritis	Parvovirosis	Y	Discharge	1	Discharge
286	Breed	F	N	0.4		Cold	Infectious	Gastroenteritis	Parvovirosis	Y	Discharge	1	Discharge
287	Breed	M	N	0.2	Not up to date	Cold	Infectious	Gastroenteritis	Parvovirosis	N	Discharge	1	Discharge
288	Breed	M	N	4.0	Up to date	Cold	Suspect	Gastroenteritis		Y	Discharge	1	
289	Mixed Breed	M	N	0.8	Up to date	Cold	Not Infectious	Distemper		Y	Discharge	1	
290	Mixed Breed	M	N	1.0	Up to date	Cold	Infectious	Distemper	Distemper	Y	Discharge	1	Discharge
291	Breed	M	N	0.2	Not up to date	Cold	Infectious	Gastroenteritis	Parvovirosis	N	Discharge	1	Discharge
292	Breed	M	N	11.0	Not up to date	Cold	Infectious	Distemper	Distemper	Y	Dead	1	Dead
293	Breed	F	N	11.0	Not up to date	Cold	Infectious	Dermatitis	Dermatophytosis	Y	Discharge	1	Discharge
294	Mixed Breed	F	Y	10.0	Not up to date	Cold	Suspect	Gastroenteritis		N	Discharge	1	
295	Breed	F	N	7.0	Up to date	Cold	Suspect	MDR		Y	Discharge	1	Discharge
296	Breed	M	N	0.4	Not up to date	Cold	Infectious	Gastroenteritis	Parvovirosis	N	Dead	1	Dead
297	Breed	M	N	0.3	Not up to date	Cold	Suspect	Gastroenteritis		N	Discharge	1	Discharge
298	Mixed Breed	F	N	0.3	Not up to date	Cold	Suspect	Distemper		Y	Discharge	1	
299	Breed	M	N	10.0	Not up to date	Cold	Not Infectious	Leptospirosis		Y	Discharge	1	Discharge
300	Breed	M	N	3.0	Not up to date	Cold	Suspect	Others		Y	Discharge	1	
301	Breed	F	N	0.6	Up to date	Cold	Infectious	Gastroenteritis	Parvovirosis	N	Discharge	1	Discharge
302	Breed	F	Y	12.0	Up to date	Cold	Suspect	Leptospirosis		N	Discharge	1	
303	Mixed Breed	F	Y	4.0	Not up to date	Cold	Suspect	Gastroenteritis		N	Discharge	1	
304	Mixed Breed	F	Y	1.0	Not up to date	Cold	Infectious	Distemper	Distemper	Y	Discharge	1	Discharge
305	Mixed Breed	M	N	13.0	Not up to date	Cold	Suspect	Leptospirosis		Y	Discharge	1	
306	Breed	M	N	11.0	Not up to date	Cold	Not Infectious	Others		Y	Discharge	1	
307	Breed	F	Y	9.0	Not up to date	Cold	Suspect	Gastroenteritis		N	Dead	1	
308	Breed	M	Y	1.0	Not up to date	Cold	Not Infectious	Distemper		N	Discharge	1	
309	Breed	F	Y	9.0	Not up to date	Cold	Suspect	Leptospirosis		N	Discharge	1	
310	Breed	M	Y	1.0		Cold	Suspect	Distemper		N	Discharge	1	
311	Mixed Breed	F	N	0.2	Not up to date	Cold	Suspect	Gastroenteritis		Y	Discharge	1	
312	Breed	M	N	10.0	Not up to date	Cold	Not Infectious	Others		Y	Discharge	1	
313	Breed	M	Y	14.0	Not up to date	Cold	Infectious	MDR	MDR	Y	Discharge	1	Discharge
314	Mixed Breed	F	Y	4.0	Up to date	Cold	Not Infectious	Leptospirosis		N	Discharge	1	
315	Mixed Breed	M	N	0.3	Not up to date	Cold	Suspect	Distemper		N	Discharge	1	Discharge
316	Breed	M	N	7.0	Up to date	Cold	Infectious	Leptospirosis	Leptospirosis	Y	Discharge	1	Discharge
317	Breed	M	N	7.0	Not up to date	Cold	Suspect	Gastroenteritis		N	Discharge	1	Discharge
318	Breed	M	N	1.0	Not up to date	Cold	Infectious	Leptospirosis	Leptospirosis	N	Dead	1	Dead
319	Breed	F	N	9.0	Not up to date	Cold	Infectious	Leptospirosis	Leptospirosis	N	Dead	1	Dead
320	Mixed Breed	M	N	14.0	Not up to date	Warm	Infectious	MDR	MDR	Y	Dead	1	Dead

321	Breed	F	N	6.0	Not up to date	Warm	Not Infectious	Leptospirosis		N	Discharge	1	
322	Mixed Breed	F	Y	14.0	Up to date	Warm	Not Infectious	Leptospirosis		N	Discharge	1	
323	Breed	M	N	7.0	Not up to date	Warm	Suspect	Gastroenteritis		Y	Discharge	1	
324	Breed	M	N	0.3	Not up to date	Warm	Infectious	Gastroenteritis	Parvovirosis	N	Discharge	1	Discharge
325	Mixed Breed	F	Y	11.0	Not up to date	Warm	Suspect	Gastroenteritis		N	Discharge	1	
326	Breed	M	N	4.0	Up to date	Warm	Infectious	MDR	MDR	Y	Discharge	1	Dead
327	Breed	F	Y	9.0		Warm	Suspect	Leptospirosis		N	Discharge	1	
328	Breed	M	Y	8.0	Not up to date	Warm	Suspect	Leptospirosis		N	Discharge	1	
329	Mixed Breed	F	N	0.3	Not up to date	Warm	Infectious	Gastroenteritis	Parvovirosis	N	Discharge	1	Discharge
330	Breed	F	N	2.0	Up to date	Warm	Not Infectious	Leptospirosis		Y	Discharge	1	
331	Breed	F	N	1.0	Up to date	Warm	Suspect	Distemper		N	Discharge	1	Discharge
332	Breed	M	N	0.1	Not up to date	Warm	Infectious	Gastroenteritis	Parvovirosis	N	Discharge	1	Discharge
333	Breed	F	N	0.1	Not up to date	Warm	Infectious	Gastroenteritis	Parvovirosis	N	Discharge	1	Discharge
334	Breed	M	N	0.1	Not up to date	Warm	Infectious	Gastroenteritis	Parvovirosis	N	Discharge	1	Discharge
335	Breed	M	N	12.0	Not up to date	Warm	Suspect	Leptospirosis		Y	Dead	1	
336	Mixed Breed	F	Y	2.0	Up to date	Warm	Not Infectious	Leptospirosis		Y	Discharge	1	
337	Mixed Breed	F	N	0.3	Not up to date	Warm	Infectious	Gastroenteritis	Parvovirosis	N	Dead	1	Dead
338	Breed	M	N	9.0	Not up to date	Warm	Not Infectious	Distemper		N	Discharge	1	
339	Breed	M	N	6.0		Warm	Suspect	Others		N	Discharge	1	
340	Breed	M	Y	15.0	Not up to date	Warm	Infectious	Leptospirosis	Leptospirosis	Y	Dead	1	Dead
341	Mixed Breed	M	N	17.0		Warm	Suspect	Others		Y	Discharge	1	
342	Mixed Breed	F	N	1.0	Not up to date	Warm	Infectious	Gastroenteritis	Parvovirosis	N	Discharge	1	Discharge
343	Breed	F	N	12.0	Not up to date	Warm	Not Infectious	Leptospirosis		Y	Discharge	1	
344	Mixed Breed	F	N	0.7	Not up to date	Warm	Infectious	Gastroenteritis	Parvovirosis	N	Discharge	1	Discharge
345	Mixed Breed	M	N	0.2	Not up to date	Warm	Infectious	Gastroenteritis	Parvovirosis	N	Discharge	1	Discharge
346	Breed	M	Y	11.0	Not up to date	Warm	Suspect	Others		Y	Dead	1	
347	Breed	M	N	0.3	Not up to date	Warm	Infectious	Gastroenteritis	Parvovirosis	N	Dead	1	Dead
348	Breed	M	N	0.9	Not up to date	Warm	Infectious	Gastroenteritis	Parvovirosis	N	Discharge	1	Discharge
349	Breed	F	Y	5.0	Not up to date	Warm	Not Infectious	Leptospirosis		N	Discharge	1	
350	Breed	M	N	8.0	Not up to date	Warm	Suspect	Leptospirosis		Y	Dead	1	
351	Mixed Breed	F	N	0.2	Not up to date	Warm	Infectious	Leptospirosis	Leptospirosis	N	Dead	1	Dead
352	Breed	M	N	7.0	Not up to date	Warm	Suspect	Leptospirosis		Y	Discharge	1	
353	Breed	M	N	5.0	Not up to date	Warm	Infectious	Leptospirosis	Leptospirosis	Y	Discharge	1	Discharge
354	Breed	M	N	0.8	Not up to date	Warm	Infectious	Gastroenteritis	Parvovirosis	N	Discharge	1	Discharge
355	Breed	M	N	10.0	Not up to date	Warm	Suspect	Leptospirosis		Y	Dead	1	
356	Mixed Breed	M	N	0.2	Not up to date	Warm	Infectious	Gastroenteritis	Parvovirosis	N	Discharge	1	Discharge
357	Mixed Breed	M	N	0.3	Not up to date	Warm	Infectious	Gastroenteritis	Parvovirosis	N	Discharge	1	Discharge
358	Mixed Breed	F	N	14.0	Not up to date	Warm	Infectious	Gastroenteritis	Parvovirosis	Y	Discharge	1	Discharge
359	Breed	F	Y	10.0	Not up to date	Warm	Not Infectious	Leptospirosis		Y	Discharge	1	
360	Breed	F	N	0.1	Not up to date	Warm	Infectious	Gastroenteritis	Parvovirosis	N	Discharge	1	Discharge
361	Mixed Breed	M	N	0.3		Warm	Infectious	Gastroenteritis	Parvovirosis	Y	Dead	1	Dead
362	Breed	F	N	0.2	Not up to date	Warm	Infectious	Gastroenteritis	Parvovirosis	N	Discharge	1	Discharge
363	Mixed Breed	M	N	0.4	Not up to date	Warm	Infectious	Gastroenteritis	Parvovirosis	Y	Discharge	1	Discharge
364	Mixed Breed	F	Y	10.0	Not up to date	Warm	Not Infectious	Leptospirosis		Y	Discharge	1	
365	Mixed Breed	M	N	8.0	Not up to date	Warm	Suspect	Others		N	Discharge	1	
366	Breed	F	N	0.2	Not up to date	Cold	Infectious	Distemper	Distemper	Y	Discharge	1	Discharge
367	Mixed Breed	M	Y	3.0	Not up to date	Cold	Infectious	Leptospirosis	Leptospirosis	N	Dead	1	Dead
368	Mixed Breed	F	N	8.0		Cold	Infectious	Leptospirosis	Leptospirosis	N	Dead	1	Dead
369	Breed	F	N	8.0	Not up to date	Cold	Suspect	MDR		Y	Discharge	1	
370	Breed	M	N	11.0	Up to date	Cold	Not Infectious	Leptospirosis		Y	Discharge	1	
371	Mixed Breed	M	N	0.2	Not up to date	Cold	Infectious	Gastroenteritis	Parvovirosis	N	Discharge	1	Discharge
372	Breed	M	N	4.0	Not up to date	Cold	Not Infectious	Distemper		N	Discharge	1	
373	Mixed Breed	F	Y	0.8	Not up to date	Cold	Infectious	Gastroenteritis	Parvovirosis	Y	Discharge	1	Discharge
374	Breed	F	N	6.0	Not up to date	Cold	Infectious	Leptospirosis	Leptospirosis	N	Dead	1	Dead
375	Breed	F	N	0.2	Not up to date	Cold	Infectious	Gastroenteritis	Parvovirosis	N	Discharge	1	Discharge
376	Breed	F	N	0.1	Not up to date	Cold	Infectious	Gastroenteritis	Parvovirosis	N	Discharge	1	Discharge
377	Breed	M	Y	12.0	Not up to date	Cold	Infectious	Leptospirosis	Leptospirosis	Y	Discharge	1	Discharge
378	Mixed Breed	M	Y	13.0	Not up to date	Cold	Suspect	Leptospirosis		Y	Discharge	1	
379	Mixed Breed	F	N	3.0	Not up to date	Cold	Suspect	Leptospirosis		Y	Discharge	1	
380	Breed	M	N	0.2	Not up to date	Cold	Infectious	Gastroenteritis	Parvovirosis	Y	Discharge	2	Dead
381	Mixed Breed	F	Y	16.0		Cold	Infectious	MDR	MDR	Y	Dead	1	Dead
382	Breed	F	N	0.2	Not up to date	Cold	Infectious	Gastroenteritis	Parvovirosis	Y	Discharge	2	Discharge
383	Mixed Breed	M	N	3.0	Not up to date	Cold	Suspect	Leptospirosis		Y	Discharge	1	
384	Breed	F	Y	7.0	Not up to date	Cold	Infectious	Leptospirosis	Leptospirosis	Y	Discharge	1	Discharge
385	Breed	M	Y	12.0	Not up to date	Cold	Suspect	Leptospirosis		Y	Discharge	1	
386	Breed	F	N	1.0	Not up to date	Cold	Suspect	Leptospirosis		N	Discharge	1	
387	Mixed Breed	M	N	16.0	Not up to date	Cold	Infectious	Dermatitis	Sarcoptic Scabies	Y	Discharge	1	Discharge
388	Breed	M	N	6.0	Up to date	Cold	Infectious	Leptospirosis	Leptospirosis	Y	Discharge	1	Discharge
389	Breed	M	N	3.0	Not up to date	Cold	Not Infectious	Leptospirosis		Y	Discharge	1	
390	Mixed Breed	F	N	7.0	Not up to date	Cold	Not Infectious	Leptospirosis		Y	Discharge	1	
391	Breed	M	N	0.2	Not up to date	Cold	Infectious	Gastroenteritis	Parvovirosis	N	Discharge	1	Discharge
392	Breed	F	N	0.6	Not up to date	Cold	Infectious	Gastroenteritis	Parvovirosis	N	Discharge	1	Discharge
393	Breed	F	Y	12.0		Cold	Not Infectious	MDR		Y	Discharge	1	
394	Breed	M	N	0.2	Not up to date	Cold	Infectious	Gastroenteritis	Parvovirosis	N	Dead	1	Dead
395	Mixed Breed	F	N	18.0	Up to date	Cold	Infectious	Gastroenteritis	Cryptosporidium	Y	Discharge	1	Discharge
396	Mixed Breed	F	N	0.2	Not up to date	Cold	Infectious	Gastroenteritis	Parvovirosis	Y	Discharge	1	Discharge
397	Mixed Breed	F	N	0.2	Not up to date	Cold	Infectious	Gastroenteritis	Parvovirosis	N	Discharge	1	Discharge
398	Mixed Breed	F	Y	3.0	Up to date	Cold	Infectious	Leptospirosis	Leptospirosis	N	Dead	1	Dead
399	Breed	M	Y	11.0	Not up to date	Cold	Not Infectious	Leptospirosis		Y	Discharge	1	
400	Breed	F	Y	6.0	Not up to date	Cold	Infectious	Leptospirosis	Leptospirosis	Y	Discharge	1	Discharge
401	Mixed Breed	F	N	8.0	Up to date	Cold	Suspect	Leptospirosis		N	Discharge	1	
402	Mixed Breed	M	Y	6.0	Not up to date	Cold	Not Infectious	Leptospirosis		Y	Dead	1	
403	Breed	M	N	8.0	Not up to date	Cold	Not Infectious	Leptospirosis		Y	Dead	1	
404	Breed	F	Y	12.0	Not up to date	Cold	Not Infectious	Leptospirosis		Y	Discharge	1	
405	Mixed Breed	F	N	4.0	Not up to date	Cold	Suspect	Others		Y	Discharge	1	
406	Mixed Breed	M	N	8.0	Not up to date	Cold	Infectious	Leptospirosis	Leptospirosis	N	Dead	1	Dead
407	Breed	M	Y	13.0	Up to date	Cold	Infectious	Dermatitis	Dermatophytosis	Y	Dead	1	Dead
408	Mixed Breed	M	N	8.0	Not up to date	Cold	Infectious	Leptospirosis	Leptospirosis	Y	Discharge	1	Discharge
409	Mixed Breed	M	N	0.2	Not up to date	Warm	Suspect	Gastroenteritis		Y	Dead	1	
410	Breed	M	N	3.0	Not up to date	Warm	Suspect	Distemper		Y	Discharge	1	
411	Breed	F	Y	10.0	Not up to date	Warm	Not Infectious	Leptospirosis		Y	Discharge	1	
412	Mixed Breed	F	N	0.1	Not up to date	Warm	Infectious	Gastroenteritis	Parvovirosis	N	Dead	1	Dead
413	Breed	F	N	7.0	Not up to date	Warm	Infectious	Leptospirosis	Leptospirosis	N	Dead	1	Dead
414	Mixed Breed	M	N	0.2	Not up to date	Warm	Infectious	Gastroenteritis	Parvovirosis	N	Discharge	1	Discharge
415	Breed	F	N	6.0	Not up to date	Warm	Suspect	MDR		Y	Discharge	1	
416	Breed	M	N	3.0	Up to date	Warm	Infectious	Dermatitis	Dermatophytosis	Y	Discharge	1	Discharge
417	Mixed Breed	M	Y	15.0		Warm	Suspect	Gastroenteritis		N	Discharge	1	Discharge
418	Breed	M	N	0.2	Not up to date	Warm	Infectious	Gastroenteritis	Parvovirosis	Y	Dead	1	Dead
419	Breed	M	N	7.0	Not up to date	Warm	Suspect	Leptospirosis		Y	Discharge	1	
420	Mixed Breed	M	N	4.0	Not up to date	Warm	Not Infectious	Distemper		Y	Discharge	1	
421	Breed	F	Y	12.0	Up to date	Warm	Infectious	MDR	MDR	Y	Discharge	1	Dead
422	Breed	F	Y	7.0	Not up to date	Warm	Not Infectious	Leptospirosis		Y	Discharge	1	
423	Mixed Breed	M	N	15.0	Not up to date	Warm	Suspect	Others		Y	Dead	1	
424	Breed	F	Y	3.0	Not up to date	Warm	Not Infectious	Leptospirosis		Y	Discharge	1	
425	Breed	M	N	4.0	Not up to date	Warm	Infectious	Leptospirosis	Leptospirosis	N	Dead	1	Dead

426	Mixed Breed	M	N	5.0	Not up to date	Warm	Suspect	Leptospirosis		Y	Discharge	1	
427	Mixed Breed	M	Y	14.0	Not up to date	Warm	Suspect	Leptospirosis		Y	Discharge	1	
428	Mixed Breed	M	N	0.5	Not up to date	Warm	Infectious	Gastroenteritis	Parvovirosis	Y	Discharge	1	Discharge
429	Mixed Breed	F	N	0.2	Not up to date	Warm	Infectious	Gastroenteritis	Parvovirosis	N	Discharge	1	Discharge
430	Mixed Breed	F	N	7.0	Not up to date	Warm	Suspect	Leptospirosis		Y	Discharge	1	
431	Breed	F	N	2.0	Up to date	Warm	Infectious	MDR	MDR	Y	Discharge	1	Discharge
432	Breed	M	N	0.2	Not up to date	Warm	Suspect	Gastroenteritis		N	Discharge	1	
433	Breed	F	N	11.0	Not up to date	Warm	Not Infectious	Leptospirosis		Y	Discharge	1	
434	Breed	M	N	0.3	Not up to date	Warm	Suspect	Gastroenteritis		Y	Discharge	1	
435	Breed	M	N	7.0	Not up to date	Warm	Not Infectious	Leptospirosis		Y	Discharge	1	
436	Mixed Breed	M	N	0.2	Not up to date	Warm	Suspect	Gastroenteritis		Y	Discharge	1	
437	Mixed Breed	M	N	12.0	Not up to date	Warm	Suspect	Others		Y	Discharge	1	
438	Breed	M	N	0.3	Not up to date	Warm	Infectious	Gastroenteritis	Parvovirosis	Y	Discharge	1	Discharge
439	Breed	M	N	9.0		Warm	Infectious	MDR	MDR	Y	Discharge	1	Discharge
440	Mixed Breed	M	N	0.3	Not up to date	Warm	Infectious	Gastroenteritis	Parvovirosis	N	Discharge	1	Discharge
441	Breed	F	N	0.3	Not up to date	Warm	Infectious	Gastroenteritis	Parvovirosis	Y	Discharge	1	Discharge
442	Mixed Breed	F	Y	1.0	Not up to date	Warm	Suspect	Others		N	Discharge	1	
443	Breed	M	N	9.0	Not up to date	Warm	Suspect	Leptospirosis		Y	Dead	1	
444	Breed	F	Y	14.0	Not up to date	Warm	Suspect	Leptospirosis		Y	Discharge	1	
445	Mixed Breed	M	N	14.0	Up to date	Warm	Not Infectious	Leptospirosis		Y	Discharge	1	
446	Mixed Breed	F	Y	8.0	Up to date	Warm	Suspect	Gastroenteritis		Y	Discharge	1	
447	Mixed Breed	M	N	0.3	Not up to date	Warm	Infectious	Gastroenteritis	Parvovirosis	Y	Discharge	1	Discharge
448	Breed	F	Y	5.0	Up to date	Warm	Suspect	Leptospirosis		N	Discharge	1	
449	Breed	M	N	13.0	Not up to date	Warm	Suspect	Others		Y	Discharge	1	
450	Breed	M	N	7.0	Not up to date	Warm	Infectious	Leptospirosis	Leptospirosis	Y	Discharge	1	Discharge
451	Breed	F	Y	14.0	Not up to date	Warm	Not Infectious	Leptospirosis		Y	Discharge	1	
452	Mixed Breed	F	N	1.0	Not up to date	Warm	Suspect	Leptospirosis		Y	Discharge	1	
453	Breed	M	N	0.2	Not up to date	Cold	Infectious	Gastroenteritis	Parvovirosis	N	Discharge	1	Discharge
454	Mixed Breed	M	N	3.0	Not up to date	Cold	Suspect	Gastroenteritis		N	Discharge	1	
455	Mixed Breed	M	N	16.0	Not up to date	Cold	Suspect	Others		Y	Discharge	1	
456	Mixed Breed	F	Y	9.0	Up to date	Cold	Not Infectious	Leptospirosis		Y	Discharge	1	
457	Breed	M	N	4.0	Not up to date	Cold	Suspect	Others		Y	Discharge	1	
458	Mixed Breed	M	N	7.0	Not up to date	Cold	Suspect	Others		Y	Discharge	1	
459	Mixed Breed	M	N	1.0	Not up to date	Cold	Suspect	Leptospirosis		Y	Discharge	1	
460	Mixed Breed	M	Y	11.0	Not up to date	Cold	Suspect	Leptospirosis		Y	Dead	1	
461	Mixed Breed	M	Y	10.0	Up to date	Cold	Infectious	MDR	MDR	Y	Dead	1	Dead
462	Breed	F	N	0.1	Not up to date	Cold	Suspect	Gastroenteritis		Y	Discharge	1	
463	Breed	M	N	7.0	Up to date	Cold	Suspect	Gastroenteritis		N	Discharge	1	
464	Mixed Breed	F	Y	12.0	Up to date	Cold	Not Infectious	Leptospirosis		Y	Discharge	1	
465	Breed	M	N	8.0	Up to date	Cold	Not Infectious	Leptospirosis		Y	Discharge	1	
466	Breed	M	N	10.0	Not up to date	Cold	Suspect	Others		Y	Dead	1	
467	Breed	F	Y	8.0	Not up to date	Cold	Suspect	Leptospirosis		Y	Dead	1	
468	Mixed Breed	M	N	6.0	Not up to date	Cold	Suspect	Others		Y	Discharge	1	
469	Mixed Breed	M	N	4.0	Not up to date	Cold	Suspect	Gastroenteritis		Y	Discharge	1	
470	Mixed Breed	M	N	0.3	Not up to date	Cold	Infectious	Gastroenteritis	Parvovirosis	Y	Discharge	1	Discharge
471	Breed	M	N	0.3	Not up to date	Cold	Infectious	Gastroenteritis	Parvovirosis	N	Discharge	1	Discharge
472	Breed	M	N	0.8	Not up to date	Cold	Infectious	Gastroenteritis	Parvovirosis	Y	Discharge	1	Discharge
473	Mixed Breed	F	N	0.3	Not up to date	Cold	Suspect	Others		Y	Discharge	1	
474	Breed	F	N	0.3	Not up to date	Cold	Suspect	Gastroenteritis		N	Discharge	1	
475	Breed	F	N	10.0	Not up to date	Cold	Infectious	MDR	MDR	Y	Discharge	3	Discharge
476	Mixed Breed	F	N	14.0	Not up to date	Cold	Infectious	MDR	MDR	Y	Dead	1	Dead
477	Breed	F	Y	8.0	Not up to date	Cold	Infectious	Leptospirosis	Leptospirosis	N	Discharge	1	Discharge
478	Breed	M	N	0.3	Not up to date	Cold	Infectious	Gastroenteritis	Parvovirosis	N	Discharge	1	Discharge
479	Breed	M	N	4.0	Up to date	Cold	Not Infectious	Leptospirosis		Y	Discharge	1	
480	Breed	F	Y	14.0	Not up to date	Cold	Suspect	Others		Y	Discharge	1	
481	Mixed Breed	F	Y	13.0	Up to date	Cold	Infectious	Leptospirosis	Leptospirosis	N	Dead	1	Dead
482	Breed	M	N	0.2	Not up to date	Cold	Infectious	Gastroenteritis	Parvovirosis	Y	Discharge	1	Discharge
483	Breed	F	N	0.2	Not up to date	Cold	Infectious	Gastroenteritis	Parvovirosis	Y	Discharge	1	Discharge
484	Breed	M	N	0.3	Not up to date	Cold	Infectious	Gastroenteritis	Parvovirosis	Y	Discharge	1	Discharge
485	Breed	F	N	12.0	Up to date	Cold	Suspect	Leptospirosis		Y	Dead	1	
486	Mixed Breed	M	N	0.2	Not up to date	Cold	Suspect	Leptospirosis		N	Discharge	1	
487	Breed	F	N	12.0	Not up to date	Cold	Suspect	Others		Y	Discharge	1	
488	Breed	F	N	6.0	Not up to date	Cold	Infectious	Gastroenteritis	Parvovirosis	N	Discharge	1	Discharge
489	Mixed Breed	F	N	0.2	Not up to date	Cold	Infectious	Gastroenteritis	Parvovirosis	Y	Discharge	1	Discharge
490	Breed	F	N	6.0	Not up to date	Cold	Infectious	Gastroenteritis	Parvovirosis	N	Discharge	1	Discharge
491	Breed	F	Y	12.0	Up to date	Cold	Suspect	MDR		Y	Discharge	1	
492	Breed	F	N	0.3	Not up to date	Cold	Suspect	Distemper		N	Discharge	1	
493	Mixed Breed	M	N	5.0	Up to date	Cold	Suspect	Leptospirosis		N	Discharge	1	
494	Mixed Breed	F	Y	13.0	Not up to date	Cold	Not Infectious	Leptospirosis		Y	Dead	1	
495	Breed	F	N	0.3	Not up to date	Cold	Infectious	Gastroenteritis	Parvovirosis	Y	Discharge	1	Discharge
496	Mixed Breed	M	N	0.2	Not up to date	Cold	Infectious	Gastroenteritis	Parvovirosis	Y	Discharge	1	Discharge
497	Breed	M	N	8.0	Not up to date	Cold	Infectious	MDR	MDR	Y	Discharge	1	Dead
498	Mixed Breed	M	Y	3.0	Not up to date	Cold	Infectious	Gastroenteritis	Parvovirosis	Y	Dead	1	Dead
499	Breed	M	Y	6.0	Not up to date	Cold	Not Infectious	Leptospirosis		Y	Discharge	1	
500	Breed	M	N	0.2	Not up to date	Cold	Suspect	Distemper		N	Discharge	1	
501	Breed	M	N	0.4	Not up to date	Cold	Infectious	Gastroenteritis	Parvovirosis	N	Discharge	1	Discharge
502	Breed	F	Y	11.0	Up to date	Cold	Suspect	Leptospirosis		Y	Discharge	1	
503	Breed	M	N	8.0	Not up to date	Cold	Suspect	Leptospirosis		Y	Discharge	1	
504	Breed	F	Y	14.0	Not up to date	Cold	Not Infectious	Leptospirosis		Y	Discharge	1	
505	Breed	F	N	10.0	Not up to date	Warm	Suspect	Gastroenteritis		N	Discharge	1	
506	Mixed Breed	F	Y	2.0	Up to date	Warm	Suspect	Others		N	Discharge	1	
507	Breed	M	N	4.0	Not up to date	Warm	Not Infectious	Leptospirosis		Y	Discharge	1	
508	Breed	F	N	0.2	Not up to date	Warm	Infectious	Gastroenteritis	Parvovirosis	N	Dead	1	Dead
509	Breed	M	N	11.0	Not up to date	Warm	Infectious	MDR	MDR	Y	Discharge	1	Discharge
510	Breed	F	Y	13.0	Not up to date	Warm	Not Infectious	Leptospirosis		Y	Discharge	1	
511	Mixed Breed	F	Y	15.0		Warm	Suspect	Others		Y	Discharge	1	
512	Breed	F	N	0.2	Not up to date	Warm	Suspect	Gastroenteritis		Y	Discharge	1	
513	Mixed Breed	M	N	0.3	Not up to date	Warm	Suspect	Gastroenteritis		Y	Discharge	1	
514	Breed	M	Y	7.0	Not up to date	Warm	Suspect	Leptospirosis		Y	Dead	1	
515	Breed	F	N	0.3	Not up to date	Warm	Infectious	Gastroenteritis	Parvovirosis	N	Discharge	1	Discharge
516	Breed	F	N	12.0	Up to date	Warm	Not Infectious	Distemper		Y	Dead	1	
517	Mixed Breed	F	N	11.0	Not up to date	Warm	Suspect	MDR		Y	Dead	1	
518	Breed	F	N	11.0	Not up to date	Warm	Suspect	Others		Y	Dead	1	
519	Breed	M	N	0.2	Not up to date	Warm	Infectious	Gastroenteritis	Parvovirosis	Y	Dead	1	Dead
520	Breed	M	N	2.0	Not up to date	Warm	Suspect	Gastroenteritis		Y	Dead	1	
521	Breed	F	N	0.3	Not up to date	Warm	Infectious	Gastroenteritis	Parvovirosis	Y	Discharge	1	Discharge
522	Breed	F	N	0.3	Not up to date	Warm	Infectious	Gastroenteritis	Parvovirosis	Y	Discharge	1	Discharge
523	Breed	F	N	0.2	Not up to date	Warm	Infectious	Gastroenteritis	Parvovirosis	N	Dead	1	Dead
524	Breed	M	N	6.0	Not up to date	Warm	Suspect	Leptospirosis		Y	Discharge	1	
525	Mixed Breed	F	N	0.2	Not up to date	Warm	Suspect	Distemper		Y	Discharge	1	
526	Breed	M	N	0.3	Not up to date	Warm	Suspect	Gastroenteritis		Y	Discharge	1	
527	Breed	F	N	0.4	Up to date	Warm	Suspect	Gastroenteritis		N	Discharge	1	
528	Mixed Breed	F	N	0.5		Warm	Suspect	Gastroenteritis		N	Discharge	1	
529	Breed	F	N	10.0	Not up to date	Warm	Suspect	Leptospirosis		N	Dead	1	
530	Breed	M	N	0.3	Not up to date	Warm	Suspect	Leptospirosis		N	Discharge	1	
531	Mixed Breed	F	Y	10.0	Not up to date	Warm	Suspect	Leptospirosis		Y	Discharge	1	
532	Mixed Breed	M	Y	13.0	Up to date	Warm	Infectious	MDR	MDR	Y	Discharge	1	Discharge
533	Breed	M	N	0.2	Not up to date	Warm	Infectious	Gastroenteritis	Parvovirosis	N	Dead	1	Dead
534	Breed	F	N	0.3	Not up to date	Warm	Infectious	Gastroenteritis	Parvovirosis	N	Discharge	1	Discharge
